# Potential Sustainable Slow-Release Fertilizers Obtained by Mechanochemical Activation of MgAl and MgFe Layered Double Hydroxides and K_2_HPO_4_

**DOI:** 10.3390/nano9020183

**Published:** 2019-02-01

**Authors:** Roger Borges, Fernando Wypych, Elodie Petit, Claude Forano, Vanessa Prevot

**Affiliations:** 1 Institut de chimie de Clermont-Ferrand (ICCF), Université Clermont Auvergne, Centre National Recherche Scientifique (CNRS), Sigma Clermont, F-63000 Clermont-Ferrand, France; 1roger.borges@gmail.com (R.B.); Elodie.PETIT@uca.fr (E.P.); 2 Departamento de Química, Universidade Federal do Paraná, Caixa Postal 19032, Curitiba—PR, CEP-81531-980, Brazil; wypych@ufpr.br

**Keywords:** slow-release fertilizers, mechanochemical activation, phosphates, potassium, layered double hydroxides

## Abstract

This study describes the behavior of potential slow-release fertilizers (SRF), prepared by the mechanochemical activation of calcined Mg_2_Al-CO_3_ or Mg_2_Fe-CO_3_ layered double hydroxides (LDH) mixed with dipotassium hydrogen phosphate (K_2_HPO_4_). The effects of LDH thermal treatment on P/K release behavior were investigated. Characterizations of the inorganic composites before and after release experiments combined X-Ray diffraction (XRD), Fourier-transform infra-red spectroscopy (FTIR), solid-state nuclear magnetic resonance (NMR), scanning electron microscopy (SEM) and energy-dispersive X-ray spectroscopy (EDX). The best release profile (<75% in 28 days and at least 75% release) was obtained for MgAl/K_2_HPO_4_ (9 h milling, 2:1 molar ratio, MR). Compared to readily used K_2_HPO_4_, milling orthophosphate into LDH matrices decreases its solubility and slows down its release, with 60% and 5.4% release after 168 h for MgAl/K_2_HPO_4_ and MgFe/K_2_HPO_4_ composites, respectively. Mechanochemical addition of carboxymethylcellulose to the LDH/K_2_HPO_4_ composites leads to a noticeable improvement of P release properties.

## 1. Introduction

Concerning plant nutrition, new products or methods to improve nutrient management have become more and more important for environmental resilience. Most inorganic fertilizers currently used in agriculture have high solubility or volatility and need to be spread over fields in excessive amounts to ensure optimal efficiency of food production. Consequently, nutrient accumulation can pollute water resources through the eutrophication of surface water reservoirs. In this scenario, economic losses are generated by these non-sustainable nutrient supplies.

Based on these issues and considering that food production is an essential activity for human life, the development of sustainable agricultural practices based on new agrochemicals is of great importance. Slow-release fertilizers (SRF) and controlled-released fertilizers (CRF) are promising chemicals formulated to deliver nutrients (e.g., N, P, K, oligoelements) under kinetically controlled conditions. They provide a nutrient supply during the desired periods of germination and plant growth, and limit losses. Such properties are of great interest from a societal and economic point of view [1]. Different strategies to produce sustainable NPK SRF are reported in the literature. Nutrients have been incorporated in various matrices either by coating [2,3], by encapsulation in polymeric matrices [4] or in emulsions [5,6], or by intercalation into layered structures [7,8]. Recently, the mechanochemical activation method [9] has been described as an efficient way to produce SRF. Clay minerals and soluble potassium phosphate salts were mixed using mechanochemical treatments and the resulting composites displayed interesting K/P release properties [10,11]. Clay minerals, natural soil components, possess a great variety of chemical compositions and layered structures reactive for the incorporation of nutrients, providing huge options for obtaining different SRF. Recent studies report on the mechanochemical treatment of kaolinite [9,10] talc, montmorillonite [12] or even chrysotile [13] conjointly with phosphate salts to produce crystalline and/or amorphous phases displaying slow release properties of interest. It is noteworthy that insoluble phosphate fractions can prevent a total P release. 

In order to prepare SRF with release performances of interest, we extended this strategy to layered double hydroxides (LDH), another family of inorganic matrices. LDH are synthetic clay minerals with anion exchange properties. The LDH structure occurs naturally as hydrotalcite mineral, and the ideal generic formula, is described as [M^2+^_1−*x*_M^3+^*_x_*(OH)_2_]*^x^*^+^(A*^n^*^−^)*_x_*_/*n*_.yH_2_O, where M^II^ and M^III^ correspond to metal cations, and A*^n^*^−^ represents anions. [14,15] LDH is built of M(OH)_6_ octahedra in a brucite-like structure, where the M^II^ to M^III^ isomorphic provides residual positive charge that is stabilized by anionic species, e.g., PO_4_^−3^, CO_3_^−2^, NO_3_^−^ located in the interlayer space. There are many methods for synthesizing LDH; e.g., coprecipitation, induced hydrolysis, sol-gel and mechanochemical activation of insoluble precursors [16,17].

On one hand, the use of LDH has been investigated for environmental remediation and phosphate adsorption [18,19]. For instance, Luengo et al. [20] studied LDH matrices synthesized by the coprecipitation method for phosphate adsorption and the results showed these to be promising environmentally friendly materials for use in aquatic systems. On the other hand, it was reported that it is possible to take advantage of the LDH anionic exchange properties to develop SRF [7,21,22,23,24]. For instance, Everaert et al. [23] reported that hydrogenophosphate intercalated MgAl was up to 4.5 times more efficient than a soluble phosphate-based fertilizer for use in acidic soils. Hatami et al. [24] also reported some important assays on adsorption and desorption of phosphate using ZnAl LDH matrix, in simulated soil solution. The results provided evidence of a partial reversible release of phosphate with a percentage of desorption lower than 35% in 0.03 mol L^−1^ KNO_3_ solution after 8 h. 

In this work, SRF were prepared by mechanochemical reaction of various mixtures of calcined LDH and K_2_HPO_4_. Mg_2_Al(OH)_6_(CO_3_)_0.5_.3H_2_O and Mg_2_Fe(OH)_6_(CO_3_)_0.5_.2.5H_2_O LDH and K_2_HPO_4_ were used as sources of Mg^2+^ and K^+^ and phosphate, respectively. Our goal was to react the LDH structure with K_2_HPO_4_ by ball milling in order to obtain K-Mg-Fe-PO_4_ phases with a large panel of solubility allowing a controlled release over a long period of time. Such an approach could allow a large amount of P to be immobilized per amount of LDH. One can theoretically calculate that for a Mg_2_Al LDH/ K_2_HPO_4_ molar ratio of 1:1, a milled material with 14.6% of P should typically be obtained, compared to 5.6% of P in the case of a hybrogenophosphate intercalated Mg_2_Al matrix. The influence of MgAl and MgFe LDH thermal treatments on the release behavior of the different products was studied using kinetic and thermodynamic studies. The effect of the addition of a biopolymer, the carboxymethylcellulose (CMC), during the mechanochemical process on the release profile was also investigated.

## 2. Materials and Methods 

### 2.1. Preparation of Slow-Release Fertilizers

Mg_2_Al(OH)_6_(CO_3_)_0.5_.3H_2_O (MgAl) and Mg_2_Fe(OH)_6_(CO_3_)_0.5_.2.5H_2_O (MgFe) LDH were synthesized by the coprecipitation method as reported in the literature [14]. Briefly, aqueous solutions of Mg^2+^ (0.66 mol L^−1^) and M^3+^ (Al^3+^, Fe^3+^) (0.33 mol L^−1^) nitrate salts, with a Mg^2+^:M^3+^ molar ratio equal to 2:1, and mixed Na_2_CO_3_ (0.50 mol L^−1^)/NaOH (2.00 mol L^−1^) solution were simultaneously added at a constant rate (5 mL/h) into an aqueous solution whose pH was automatically maintained at 10.5. After the addition was completed, the precipitates were collected after repeated cycles of centrifugation and washing. Samples were then dried at room temperature. The as-prepared LDH were calcined at different temperatures (200 °C, 300 °C, 400 °C and 500 °C) for 4 h in air.

### 2.2. Mechanochemical Treatment

A planetary ball mill, PM 200 model from Retsch equipped with a 50 mL agate jar and ten agate balls of 10 mm was used for this study (rate 450 rpm). The mechanochemical treatment was investigated in three steps. Firstly, the calcined LDH materials were milled with K_2_HPO_4_ with a LDH/ K_2_HPO_4_ molar ratio (MR) equal to 1:2 for 9 h to study the effect of the thermal treatment on the performances of SRF. Secondly, systematic mechanochemical treatments were performed on LDH and K_2_HPO_4_ mixtures with different molar ratios (MR) of LDH/ K_2_HPO_4_ (1:2, 1:1, 2:1) and different milling times (3, 6, and 9 h) according to the experimental plan (2^2^), with the central point in triplicate. The results showed that the MgAl_200_/K_2_HPO_4_ system gave the best results for the 1:2 molar ratio, while for the MgFe_200_/K_2_HPO_4_ system, the best release was obtained for a 2:1 molar ratio (results not shown). 

From the sets of milling and K/P release experiments, LDH/K_2_HPO_4_ selected materials were submitted to subsequent ball milling incorporation of carboxymethylcellulose (CMC). The CMC was incorporated at a weight content of 20% of the total mass of the solid. The addition was performed following three different protocols (Table 1). The first involved the addition of CMC with a previously milled LDH/K_2_HPO_4_ composite, followed by a final grinding of 3 h. The second method involved the ball-milling of CMC and LDH/ K_2_HPO_4_ both previously ground for 3 h and 9 h, respectively. Finally, the latter method involved one-step milling of CMC/LDH/K_2_HPO_4_ mixtures for 9 h.

### 2.3. Release Experiments 

The release experiments were performed under isothermal conditions at 10 °C, 25 °C and 50 °C by adding 25 mg of SRF into 10 mL of deionized water, simulating flooded crops, i.e., with a higher release rate than when applied to the soil. At different times, ranging from 1 h to 31 days, solids were separated and concentrations of released P and K in supernatants were analyzed by ICP-AES. Solids and solutions obtained after 1 h and 168 h were used for further detailed experimental investigations.

### 2.4. Characterization Techniques 

Powder X-ray diffraction (PXRD) data were obtained using a Panalytical X’pert diffractometer with a CuKα radiation (λ = 1.54155 Å). Fourier-transform infrared spectroscopy (FTIR) measurements were carried out on solid products using a Thermo Nicolet 5700 spectrometer, employing KBr tablets at a mass ratio of 1:100 (sample:KBr) with a resolution of 4 cm^−1^ and accumulation of 64 scans. The solid-state ^27^Al, ^31^P and ^29^Si NMR spectra were acquired using a Bruker AVANCE 300 spectrometer operating at 7.05 Tesla, equipped with a 4 mm zirconia multinuclear solids probe and magic angle spinning at 12 kHz. Thermagravimetric analysis (TGA) curves and differential thermal analysis (DTA), used to calculate the water content in the LDH precursors, were obtained with a TG-DTA SETSYS Evolution analyzer from SETARAM, using 150 μL alumina crucibles and a heating rate of 5 °C min^−1^ under air flow of 50 mL min^−1^. 

The scanning electron microscopic (SEM) images were obtained using a Cambridge Scan 360 SEM operating at 1 kV and a Zeiss supra 55 FEG-VP operating at 3 keV. The samples were mounted on conductive carbon adhesive tabs for imaging.

## 3. Results

### 3.1. Influence of LDH Thermal Treatment on Milled Compounds and Their Release Properties

**Figure 1:**
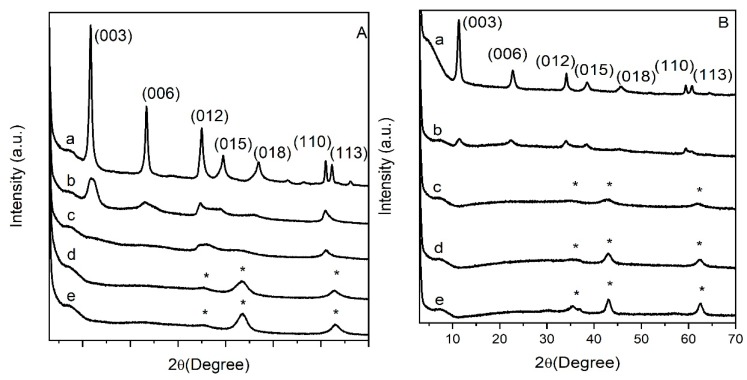
PXRD data of (**A**): MgAl (a), MgAl calcined at: 200 °C (b), at 300 °C (c), at 400 °C (d) and at 500 °C (e); and (**B**): MgFe (a), MgFe calcined at: 200 °C (b), at 300 °C (c), at 400 °C (d), and at 500 °C (e).

Powder X-ray diffraction (PXRD) patterns of pristine MgAl and MgFe LDH materials (Figure 1) confirmed the preparation of pure carbonate LDH phases crystallizing with the expected hexagonal structure (R-3m symmetry) and typical interlayer distances of 0.76 nm and 0.78 nm for MgAl-CO_3_ and MgFe-CO_3,_ respectively, consistent with the literature [25]. The presence of carbonate anions was further confirmed on the FTIR spectra by the presence of the intense band (ν_3_) at 1354 cm^−1^ (Figure S1). The first attempts to prepare SRF by ball milling K_2_HPO_4_ with as-prepared LDH were unsuccessful. The high hydration states of MgAl and MgFe LDH prevents any incorporation or reaction between LDH and phosphate salts and limits the efficiency of the milling process for SRF preparation. Consequently, LDH were dehydrated before the milling step. Thermal treatments were performed in the temperature range of LDH dehydration and dehydroxylation (200 °C–500 °C). As already reported in the literature [15], MgAl-CO_3_ LDH can undergo reversible calcination/rehydration processes when the conditions of calcination do not exceed MgO and MgAl_2_O_4_ crystallization (T < 500 °C). Interestingly, when rehydration of the amorphous phases is conducted with solutions containing anions, the LDH structure intercalated by these specific anions is then reconstructed [26]. The PXRD of calcined LDH are displayed in Figure 1A,B. MgAl and MgFe LDH structures were partially maintained after calcination at 200 °C despite a net decrease in the diffraction line intensity. At 300 °C, the diffraction lines characteristic of the LDH disappeared due to total structure collapse. For both MgAl and MgFe matrices, calcinations over 300 °C led to the formation of amorphous mixed oxides that retained a layered structuration, the so-called layered double oxides (LDO). Diffraction lines pointed at 35.4°, 43.4° and 63.0° (2θ) correspond to amorphous MgO. These structural changes were further confirmed by FTIR analysis (Figure S1). The progressive loss of the CO_3_^2−^ stretching bands (ν_3_ cm^−1^; ν_1_ cm^−1^) and of the MO-H (ν cm^−1^) and M-(OH)_6_/OMO (ν cm^−1^) lattice vibrations of the LDH structures, and the occurrence of MO_6_ lattice bands at low energy, account for dehydroxylation/decarbonatation processes and these thermally-induced structural transformations.

**Figure 2:**
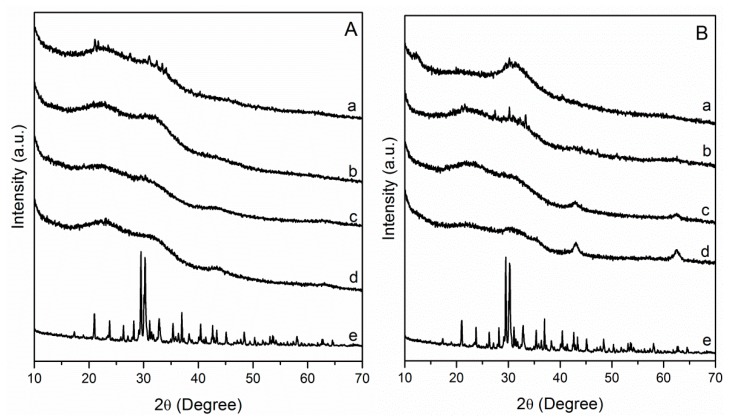
PXRD data of milled samples (9 h, 450 rpm) (**A**): MgAl_200_/K_2_HPO_4_ (a), MgAl_300_/K_2_HPO_4_ (b), MgAl_400_/K_2_HPO_4_ (c), MgAl_500_/K_2_HPO_4_ (d), K_2_HPO_4_ (e); and (**B**): MgFe_200_/K_2_HPO_4_ (a), MgFe_300_/K_2_HPO_4_ (b), MgFe_400_/K_2_HPO_4_ (c), MgFe_500_/K_2_HPO_4_ (d), K_2_HPO_4_ (e) with LDH/K_2_HPO_4_ MR 1:2.

Then the various calcined LDH were tested for their reactivity with K_2_HPO_4_ and the effect of milling treatment was evaluated using PXRD analysis in order to relate structural changes to release behaviors (Figure 2). When pure K_2_HPO_4_ was submitted to a similar grinding treatment, no structural transformation was observed. After milling LDH/K_2_HPO_4_ mixtures, X-ray patterns of pristine solids disappeared, and all residual solid composites resulted in an amorphous phase. The diffraction feature of K_2_HPO_4_ was no longer visible; the grinding made it totally react with calcined LDH, leading to unidentified structures. For composites ground using MgAl and MgFe LDH calcined at low temperatures (200 °C, 300 °C), a new phase emerged associated with diffraction lines of low intensity corresponding to the struvite-K phase (KMgPO_4_.6H_2_O) (ICDD 75-1076) [27] (Table S1). Additive structural information was obtained from FTIR analysis of MgAl-based compounds (Figure S2) whose spectra displayed features of well-identified vibrations. In the intermediate energy region (1000 cm^−1^–1600 cm^−1^), ν_3_ (antisymmetric stretch. of HPO_4_^2−^ and PO_4_^3−^), ν_1_ (antisymmetric stretch. HPO_4_^2−^ and PO) and ν_4_ (OPO bending) stretching bands of phosphate anions were clearly observed at 1045–1064, 980−983, and 532–540 cm^−1^ respectively, with values depending on the calcined LDH precursors. The vibration bands progressively shifted to those of pure K_2_HPO_4_. The higher the calcination temperature, the closer the bands were to K_2_HPO_4_, indicating greater reactivity of the MgAl LDH calcined at lower temperature (200 °C).

**Figure 3:**
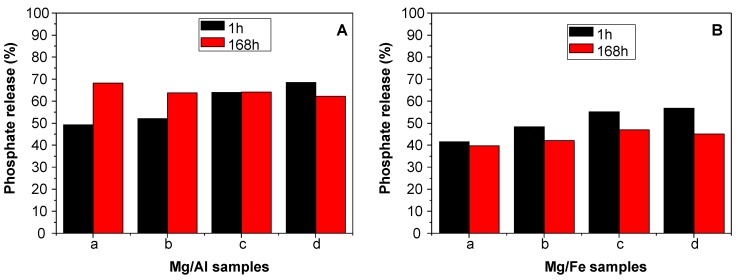
Phosphate release essays at 1 hour and 168 hours for MgAl/K_2_HPO_4_ (**A**); and MgFe/K_2_HPO_4_ (**B**) systems, with LDH calcined at: 200 °C (a), 300 °C (b), 400 °C (c) and 500 °C (d).

A preliminary study of phosphate release was performed for two release times, 1 h and 168 h (Figure 3). It is important to bear in mind that LDH have low solubility (K_SO_ (Mg_2_Al(OH)_6_(CO_3_).3H_2_O) = 25.43 [28] while K_2_HPO_4_ is totally soluble for the concentrations used in this study. First of all, it must be noted that phosphate was never fully released at the end of the experiments. Release after 1 h increased linearly with calcination temperature. Indeed, from 200 °C to 500 °C, release was improved by 39.01% and 36.80% for MgAl and MgFe, respectively. The amount of P recovery was about 8% (mean value) higher for MgAl/K_2_HPO_4_ than for MgFe/K_2_HPO_4_. After 168 h (7 days) of contact time, release did not increase compared to the earlier stage except for MgAl_200_/K_2_HPO_4_ and MgAl_300_/K_2_HPO_4_ (+19.0% and +11.74%, respectively). Unexpectedly, we even observed a decrease of phosphate leaching for MgAl_500_/K_2_HPO_4_ (−6.2%) and MgFe/K_2_HPO_4_ (−1.2%, −6.3%, −8.2%, and −11.8% for T: 200 °C, 300 °C, 400 °C, and 500 °C, respectively). A dissolution/precipitation process probably explains such results. In conclusion, most of the release was obtained in 1 h, similar to other potential CRF such as MgAl-PO_4_ LDH [7], or milled hydroxyapatite/ammonium sulfate [29]. However, the rate of P release should slow down in soils were water concentration and water diffusion, and PO_4_ mobility are reduced. Interestingly, a reserve of less soluble PO_4_ was formed either during milling or in solution, during the release experiment, allowing a longer PO_4_ release under acidic soil conditions. The remaining insoluble phosphate phases were formed either during milling or in solution, during the release experiment. According to this 2-data point kinetic study, MgAl_200_/K_2_HPO_4_ provides the best release profile, with the highest release content after 168 h and the highest release gradient from 1 h to 168 h (+19.1%). Therefore, in the following sections we focus on MgAl_200_/K_2_HPO_4_ (9 h, MR 1:2) and MgFe_200_/K_2_HPO_4_ (9 h, MR 2:1) for further investigation of SRF behavior.

**Figure 4:**
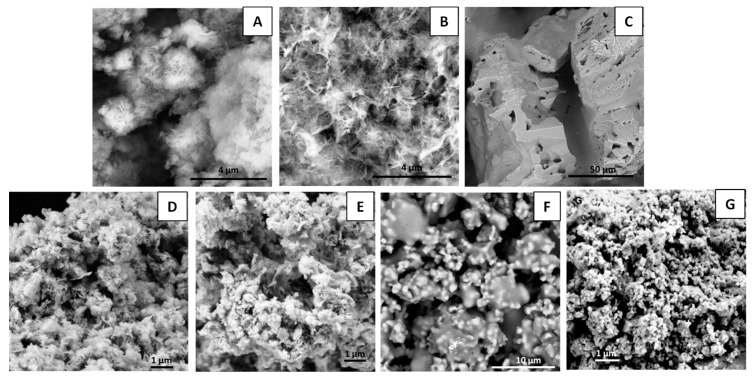
SEM images of MgAl LDH (**A**); MgFe LDH (**B**); K_2_HPO_4_ (**C**); MgAl_200_ (**D**); MgFe_200_ (**E**); MgAl_200_/K_2_HPO_4_ (**F**); and MgFe_200_/K_2_HPO_4_ (**G**).

**Figure 5:**
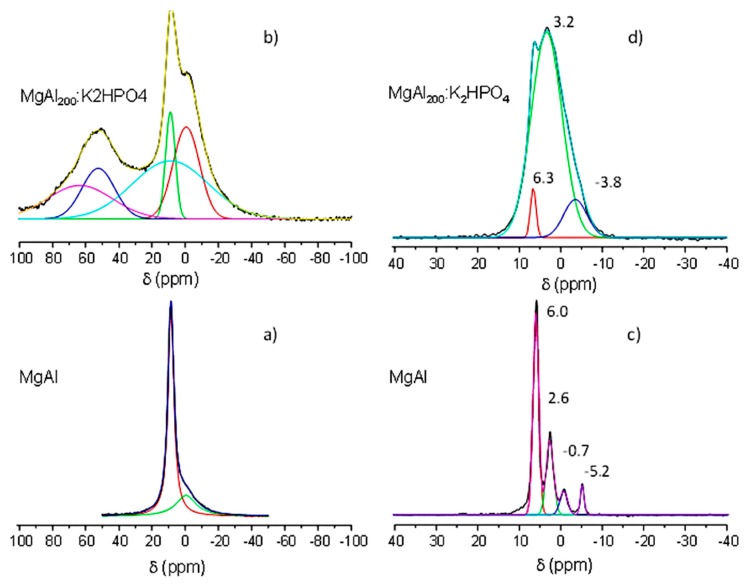
^27^Al NMR spectra of MgAl (**a**); and MgAl_200_/K_2_HPO_4_ (**b**); and ^31^P NMR spectra of K_2_HPO_4_ (**c**); and MgAl_200_/K_2_HPO_4_ (**d**).

SEM (Figure 4) and solid-state NMR (Figure 5) analyses of both MgAl_200_/K_2_HPO_4_ and MgFe_200_/K_2_HPO_4_ amorphous solids were realized in order to better understand the structural and textural changes over grinding preparation and release conditions.

In analyzing the SEM images, after calcination at 200 °C (Figure 4D,E) the LDH precursor particles appeared to be more aggregated, but the characteristic layered morphology was still evident. Pure K_2_HPO_4_ (Figure 4C) presented huge molten crystals. After mechanochemical activation, there were considerable changes in the morphologies. The image of MgAl_200_/K_2_HPO_4_ compound (Figure 4F) presents welded crystals. For the MgFe_200_/K_2_HPO_4_ image (Figure 4G), the milling step induced a reduction of the particle size while the characteristic feature of the K_2_HPO_4_ was no longer observed. It is noteworthy that both LDH based systems presented different morphologies after milling compared to the characteristic morphologies of the precursors.

The main ^27^Al NMR signals (74.4%) of MgAl (Figure 5a) pointed at 8.6 ppm is characteristic of Al^III^ cations in the octahedral environment, as expected for aluminum-containing hydrotalcite structures [30,31]. The broad but low intense peak (25.6%) centered at −0.4 ppm may be assigned to amorphous aluminum hydroxide Al(OH)_3_ as discussed by Gro Nielsen and colleagues [32]. After the milling step between MgAl-calcined and K_2_HPO_4_, the octahedral ^27^Al signal (Figure 5b) remained present [33]; however, two additional peaks appeared at δ = 52.3 ppm and δ = 63.5 ppm, typical of tetrahedral Al^3+^ sites (δ = −0.7 ppm). Tetrahedral sites arise from partial migration of Oh Al^3+^ out of the MgAl LDH layers during calcination. This evidence suggests that the mechanical activation of the MgAl_200_/K_2_HPO_4_ mixture strongly affected these two structures, leading to a diffusion of the Al^3+^ cation from Oh to Td sites. Moreover, the proportion of the amorphous Al phase increases under milling, from 25% to 87%.

The ^31^P NMR spectra of anhydrous K_2_HPO_4_ (Figure 5c) was characterized by four sharp signals located at δ = 6.0; 2.6; −0.7 and −5.2 ppm [34]. After the mechanochemical reaction between calcined MgAl and K_2_HPO_4_, the ^31^P NMR signals (Figure 5d) broadened over a large spectral width (δ = −10 to +15 ppm) due to both strong amorphization and a change in the P environment. The main signal of the free orthophosphate (δ = 6.0 ppm) decreased in favor of the high field signal characteristic of bound phosphate (δ = 3.2 ppm and δ = −3.8 ppm). We note the presence of a sharp peak at 6.3 ppm that is clearly assigned, in agreement with PXRD data, to struvite-K [35].

### 3.2. Detailed Kinetic Study

**Figure 6:**
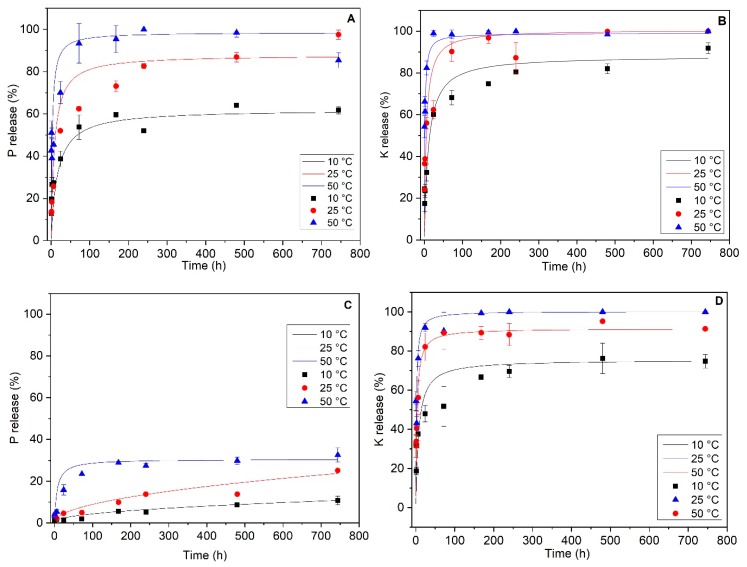
P (**A,C**) and K (**B,D**) release assays for MgAl_200_/K_2_HPO_4_ (**A,B**) and MgFe_200_/K_2_HPO_4_ (**C,D**). The symbols correspond to experimental data and the curves show the corresponding fit.

The complete kinetic release study was performed by monitoring the K and P concentrations released in solution versus time. The experiments were duplicated at three different temperatures (10 °C, 25 °C and 50 °C). Figure 6 displays the P and K released concentrations for the different systems. From these data, the plots were fitted with different kinetic models (the pseudo-first order or Lagergren model, the pseudo-second order, and the intraparticular diffusion models [36,37,38,39] that may account for the diffusion pathways of phosphate and potassium ions from the bulk and the surface of the different inorganic phases. The kinetic constants (k) were calculated from the best reliable models. Comparison of the R-squared (R^2^) regression fit factors, calculated from the fit of the linearized forms of the kinetic models and agreements between the experimental and calculated concentration values of the desorbed element at the equilibrium time (Table 2 and Table 3) allowed us to assign the best kinetic models. The best fits were obtained for a pseudo-second order kinetic diffusion process ($dq/dt=k\_2\;(q_{e}-q_{t})$, where k_2_ (g·mg^−1^·min^−1^) is the constant rate, *q_e_* (mg/g) is the total amount of molecules desorbed at equilibrium, *q* (mg/g) is the amount of molecules desorbed at *t* that accounts for a rate process limited by the rate of diffusion of the ions in the pores of the matrices [12] due to chemical interactions with the host structure. Kinetic values for P and K release of MgAl_200_/K_2_HPO_4_ experiments set at the three different temperatures are given in Table 2. Both MgAl_200_/K_2_HPO_4_ and MgFe_200_/K_2_HPO_4_ systems present results which account for a characteristic slow-release fertilizer, according to other studies in the literature; i.e., a fast release regime at the initial stage that occurs over a period of about 24 h, followed by a slow release that may continue until 744 h (the last experiment data point) and probably beyond.

Constant rate values of K and P releases were in the same range of values between 0.0702 min^−1^ and 0.519 min^−1^ even though they were not correlated with each other. Obviously, in the MgAl_200_/K_2_HPO_4_ composite, K^+^ and HPO_4_^2−^ are no longer linked in a single phase. Milling forced both ions to react separately with the LDO structure and consequently undergo release with different mechanisms, comprising dissolution and ion exchange reactions. The kinetics are strongly dependent on the temperature of the release medium. Indeed, kinetic constant (k) increased in the 10 °C–50 °C temperature domain from 0.070 min^−1^ to 0.519 min^−1^ (7.4×) and from 0.085 min^−1^ to 0.481 min^−1^ (×5.7) for K and P, respectively. As expected, dissolution and ion diffusion processes involved in the mechanisms of K and P releases were activated by thermal energy.

For the MgFe_200_/K_2_HPO_4_ system (Table 4), K release behavior was similar to MgAl_200_/K_2_HPO_4._ In either case, at temperatures of 50 °C and 25 °C (also at 10 °C for MgAl_200_/K_2_HPO_4_) 90%–100% of K was released after 744 h; but it can be observed that at 10 °C for MgFe_200_/K_2_HPO_4_, 75% of K was released after 744 h, indicating that LDO structure from the MgFe LDH structure presented more interaction with K ions. The K release process was faster than for P, probably because each one presented a different chemical interaction with LDO structure, well-fitted by a pseudo-second order model, and controlled by thermal activation. The same mechanism was reported by Borges et al. [12] for SRF prepared by ball milling K_2_HPO_4_ with montmorillonite and talc. The maximum kinetic rate was obtained at the intermediate temperature of 25 °C (1.06 min^−1^) suggesting competition between opposite mechanisms.

The release of P by the MgFe_200_/K_2_HPO_4_ solid was very different from the other kinetic behaviors. First of all, only a small fraction of loaded P was released over the duration of the experiment, with only 10.8%, 25.1% and 32.5% at 10 °C, 25 °C and 50 °C, respectively. At 50 °C, the equilibrium of release was nearly reached, while for lower thermal activations the process was far from completed. Modeling these kinetic plots gave the best results with an intra-particular diffusion mechanism at 10 °C and 25 °C. In such cases, the overall release process is controlled by one or more diffusion steps (surface, pore, grain boundary, external diffusions) and the release constant rate is limited by the overall diffusion rate given by the fit (Table 4). Clearly, phosphate diffusion through MgFe_200_/K_2_HPO_4_ particles was very slow (*k_II_* < 0.003 min^−1^ at 25 °C). This slow-down effect arises from the substitution of Al^3+^ by Fe^3+^ in the LDH. The strong reactivity between Fe^3+^ and phosphate leads to the formation of insoluble Fe/ phosphate phases and decreases the mobility of the free phosphate anions inside the fertilizer bulk.

Nearly 100% of K^+^ was released by both materials, MgAl_200_/K_2_HPO_4_ and MgFe_200_/K_2_HPO_4_, at T greater than 25 °C, while for a similar study reported for milled Montmorillonite/K_2_HPO_4_ and talc/K_2_HPO_4_ [12] K^+^ release did not exceed 70% of the loaded amounts. Indeed, clay structures are known to irreversibly fix K^+^ cations in the hexagonal holes of the structures, while no such sites exist in the LDO structure where K^+^ cations were loosely confined in mesopores.

### 3.3. Structural and Textural Characterizations of Residues upon Release

**Figure 7:**
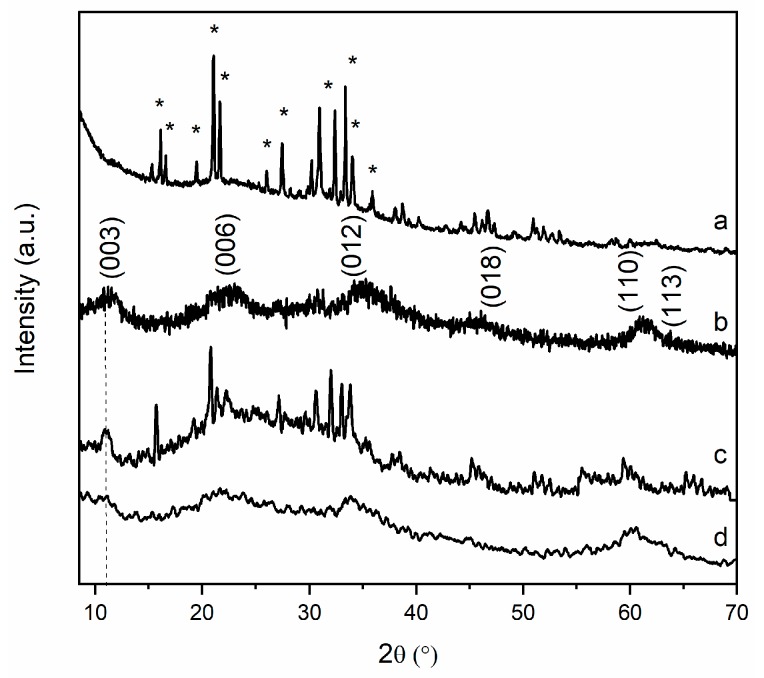
PXRD data for MgAl_200_/K_2_HPO_4_ after release time of 1 h (a) and 168 h (b), and MgFe_200_/K_2_HPO_4_ after release time of 1 h (c) and 168 h (d). * K-struvite.

To get more insight into the process involved during the release, the solid residues isolated at 1 h and 168 h (7 days) were characterized. The PXRD data of MgAl_200_/K_2_HPO_4_ and MgFe_200_/K_2_HPO_4_ residues (Figure 7) showed major changes between 1 h and 168 h. In the early stage of release (<1 h), K-struvite was formed concomitantly with the LDH structure reconstruction. This rapid phase transformation promotes the fast K and P release rates. K-struvite (KMgPO_4_·6H_2_O) is the structural analogue of the NH_4_-struvite which is used as a bioavailable source of Mg, K and P nutrients for plant growth. The solubility of NH_4_-struvite has been the subject of many studies [40]. Data from the literature report pKs values from 9.41 to 13.15. Kinetic behaviors may be quite fast depending on the crystal morphology [41]. We observed that after 168 h of release there was no more crystallized K-struvite in the solid residue, and then LDH-rebuild remained as the insoluble residue. Consequently, LDH layer composition might undergo noticeable modifications, as part of Mg^2+^ must be transferred from the initial calcined LDH to the struvite phase.

**Figure 8:**
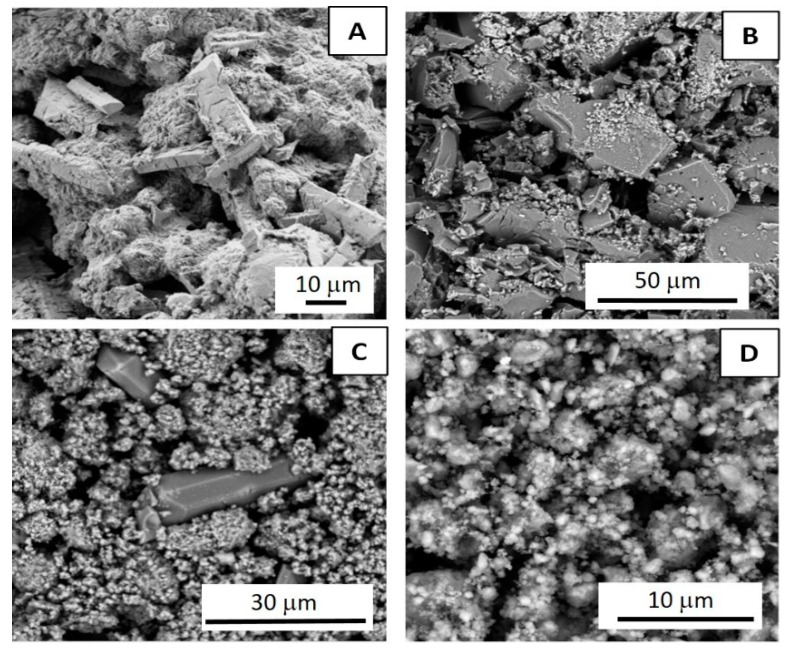
SEM images of MgAl_200_/K_2_HPO_4_ residues after 1 h (**A**) and 168 h (**B**); and of MgFe_200_/K_2_HPO_4_ residues after 1 h (**C**) and 168 h (**D**).

Comparisons of SEM images (Figure 8) of precursors and solid residues for both MgAl_200_/K_2_HPO_4_ and MgFe_200_/K_2_HPO_4_ systems, showed strong morphological changes, in good agreement with PXRD data. K-struvite presented as large rod-like crystals beside nanoparticles probably composed of amorphous mixed oxides from LDH. As discussed for PXRD results, SEM images for the residues after 168 h of release (Figure 8B,D) no longer presented K-struvite crystals. For the Mg/Al system there were huge compact crystals, probably composed of the aggregation of small LDH particles. In the case of the Mg/Fe system, some aggregated particles with ill-defined morphology were apparent.

**Figure 9:**
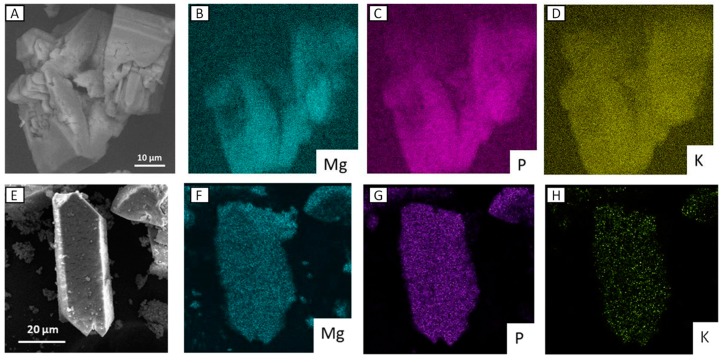
EDX images of MgAl_200_/K_2_HPO_4_ (**A**–**D**) and MgFe_200_/K_2_HPO_4_ (**E**–**H**) residues after 1 h.

Energy-dispersive X-ray spectroscopy (EDX) mapping was recorded for the two residues after 1 h of release assay (Figure 9). Analyses were focused on the large crystal that appeared under release. Struvite-K was clearly identified by the concomitant presence of Mg, P and K, homogeneously distributed over the crystals. No Al or Fe were detected in this region.

**Figure 10:**
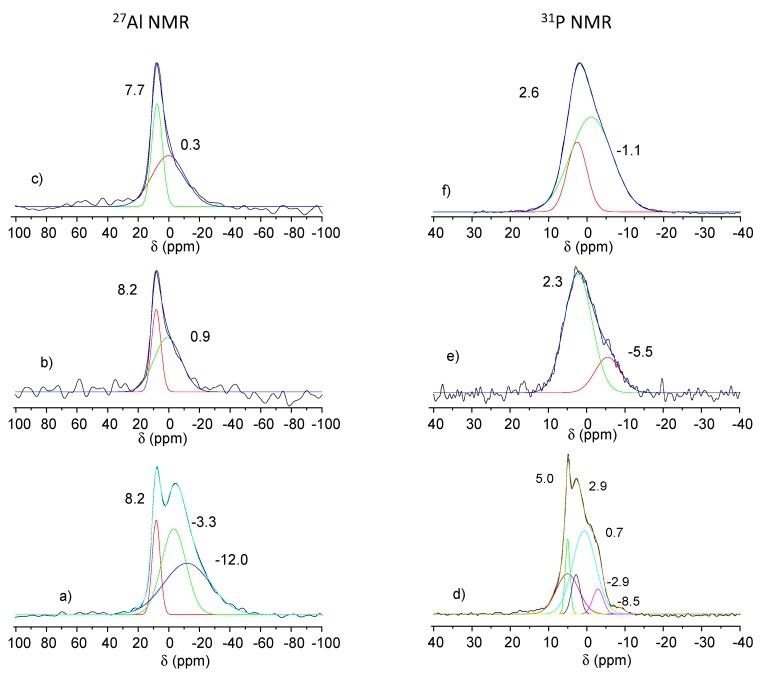
^27^Al SSNMR spectra of MgAl_200_/K_2_HPO_4_ residues after 1 h (**a**); 7 days (**b**); 31 days (**c**); and ^31^P SSNMR spectra of MgAl_200_/K_2_HPO_4_ residues after 1 h (**d**); 7 days (**e**); 31 days (**f**).

These structural changes were also confirmed by ^27^Al NMR analysis of the various solid residues. From the ^27^Al NMR spectra (Figure 10), the tetrahedral Al(IV) (52.3 and 63.5 ppm) disappeared after 1 h water contact, with the octahedral Al(VI) site remaining, in agreement with the reconstruction of the structure of Mg_2_Al LDH phase (Figure 10a). At the same time, two broad signals appeared at δ = −3.3 ppm and −12.0 ppm after 1 h of release and then disappeared for a longer period of contact time (7 and 31 days). Such an upfield signal may be assigned to amorphous aluminum hydroxide (δ = −3.3 ppm) and Al(VI)-PO_4_ (δ = −12.0 ppm) species that form due to Mg diffusion from LDH layer into struvite. For residues at 7 and 31 days, ^27^Al NMR spectra are characteristic of mixture of MgAl LDH and amorphous Al(OH)_3_ (Figure 10b,c). The ^31^P NMR spectra of residue at 1 h (Figure 10d) can be resolved into five components. Compared to the ^31^P NMR spectra of the MgAl_200_/K_2_HPO_4_ precursor, three well-identified signals at 5.0, 0.7 and −2.9 ppm occurred concomitantly. These components, which disappeared after 7 and 31 days of release, must be related to struvite-K, according to XRD analysis, even though they appeared to have shifted slightly upfield compared to the chemical shift of struvite. Residue derived from the dissolution of struvite-K (7 and 31 days of release) contained phosphate groups with a wide variety of chemical environments, as shown by the broad ^31^P NMR signals observed (Figure 10e,f). 

### 3.4. Characterization Results of Assays Involving the Incorporation of Carboxymethylcellulose (CMC)

Carboxymethylcellulose (CMC), an environmentally friendly polymer, has been used in CRF/SRF formulation, due to its biodegradable properties and water swelling capacity that allow control of NPK nutrient release by soil moisture conditions [42,43]. SRF based on inorganic compounds (silica, clay minerals) have been formulated with CMC in order to improve the swelling properties and mechanical strength of the SRF nanocomposites [44]. Using a similar strategy, in order to better tune the K and P release kinetics of MgAl_200_/K_2_HPO_4_ and MgFe_200_/K_2_HPO_4_ composites, and particularly to slow down the process at the earlier stage of release, CMC was used as a binder to densify the assemblies of LDH and K_2_HPO_4_ crystallites. Composites made of calcined LDH, K_2_HPO_4_ and CMC were prepared following three different methods as described in the Materials and Methods section.

PXRD data of CMC/LDH/K_2_HPO_4_ composites are shown in Figure S3. As reported by Biswal and Singh [44], milling of pure CMC does not affect its structure with a broad diffraction pattern in the region of 15° to 30° (2θ) characteristic of an amorphous biopolymer. When milled with MgAl_200_ and K_2_HPO_4_, whatever the method, the characteristic CMC broad diffraction peak was no longer observed. This indicates that under mechanochemical treatment with LDH and K_2_HPO_4_, CMC has undergone a high level of dispersion into the mixed mineral composite. Inorganic LDH particles and CMC are closely associated thanks to the charge compatibility between positive LDH layer surface and anionic CMC carboxy groups. In contrast, incorporation of CMC into the MgFe_200_/K_2_HPO_4_ mixture is less effective since the CMC wide peak is partially maintained. Again, when LDH and K_2_HPO_4_ were milled with CMC, both structures collapsed, leading to samples with a predominant amorphous state. ^13^C NMR analysis (Figure S4 and Table S2) of CMC milled alone, or with LDH_200_/K_2_HPO_4_, revealed the structural stability of the biopolymer under the various treatments. The milling with CMC led to an increase of amorphization of the matrix as shown by ^27^Al and ^31^P NMR spectra (Figure S5).

The SEM images (Figure S6) show evidence of drastic changes in CMC morphology; its long micrometric morphology was no longer observed for both ground samples [45]. These observations corroborate the XRD data, indicating that the polymer was modified by mechanochemical reaction with the LDH and K_2_HPO_4_ mixtures. When the MgAl_200_ grinding was performed, the sample presented in the form of large aggregates with a molten appearance, as already observed for the sample of MgAl_200_/K_2_HPO_4_ without CMC. For the sample involving grinding with MgFe_200_, agglomerates of small particles without molten appearance were observed.

### 3.5. Kinetics of Nutrients Release for MgAl_200_/K_2_HPO_4_ and MgFe_200_/K_2_HPO_4_ Formulated with CMC

As a first step, the influence of the three different milling methods on the release properties was investigated. The release experiments (Table 4) highlight that the milling sequences used to prepare the compounds had very little effect on K/P release. Systematically, in the earlier release stage (1 h), all MgAl_200_/K_2_HPO_4_ and MgFe_200_/K_2_HPO_4_ samples formulated with CMC displayed significantly slower release behavior compared to the previously studied composites without CMC (Figure 3), for both P and K. It must be noted that no Mg^2+^ release was observed. Mg^2+^ released after struvite-K solubilization probably reacted with amorphous hydroxides to precipitate Mg based LDH. 

After 168 h of contact with water solution (second release period), CMC/MgAl_200_/K_2_HPO_4_ samples presented a release percentage of P comparable to that of CMC-free solid (68.2%, Figure 3). According to the release percentage, the best slow-release profiles over the studied period of time were observed for CMC/ MgAl_200_/K_2_HPO_4_ obtained through Method 2. 

Interestingly, for the CMC/MgFe_200_/K_2_HPO_4_ composite, a noticeable improvement of P release behavior was obtained for CMC/MgFe_200_/K_2_HPO_4_ prepared when CMC was milled with MgFe_200_/K_2_HPO_4_ (Method 1) (Table 4). After 168 h of the experiment, P-release increased (9.9% to 33.4%) compared to MgFe_200_/K_2_HPO_4_, while K-release slightly decreased (99.4 to 82.7%), leading to it being considered as the most promising MgFe-based SRF candidate.

**Figure 11:**
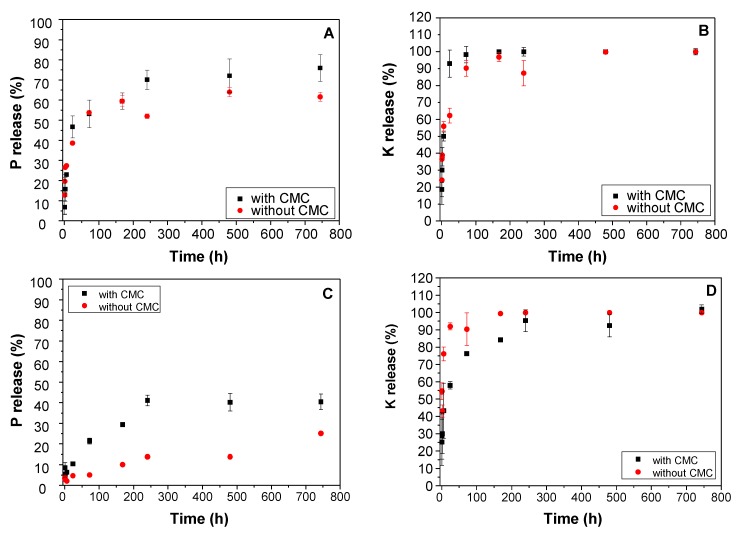
Comparison between release curves for the MgAl_200_/K_2_HPO_4_ system (Method 2) (**A,B**) and the MgFe_200_/K_2_HPO_4_ system (Method 1) (**C**,**D**) with CMC, and without CMC.

Figure 11 displays the kinetics of K and P releases (25 °C) by CMC/ MgAl_200_/K_2_HPO_4_ (Method 2) and CMC/ MgFe_200_/K_2_HPO_4_ (Method 1) composites and related CMC-free compounds (MgAl_200_/K_2_HPO_4_ and MgFe_200_/K_2_HPO_4_, respectively). Note that for the MgAl based sample, following what has already been observed for the punctual data (1 h and 168 h), with the addition of CMC, the first step starts with smaller release percentages considering the standard deviation of the tests. It is observed that for the slower stage (at 744 h) there were higher release percentages for P without significant changes for K. These are strong indications that the polymer incorporated into the material potentially influences the nutrient release behavior.

This is even more significant in relation to the release curves for the MgFe based sample. At initial release times the percentage of released P was slightly higher compared to the sample without CMC. Along the release curve, this effect becomes even more pronounced (at 744 h, without CMC 25.1%, and with CMC 40.4%), emphasizing that in this matrix the presence of CMC allowed P to be made more available, i.e., to increase the percentage of release over time. In the case of K which is highly available, the CMC led to a slight decrease in the percentage released over time for the initial release phase, and also for the intermediate release points (between 72 h and 240 h).

These results highlight that the addition of CMC in the milling process modifies the release behavior of the nutrients K and P, especially at short release time, making the products more controlled according to the demand. 

Interestingly if we compare the results obtained from LDH matrices with SRF prepared by mechanochemical activation of cationic clays such as montmorillonite and talc [12], it appears clear that in LDH-based SRF, the potassium is much more available systematically leading to 100% release at the longer time (744 h), while the P release amount can be tuned in a larger range from 25.1% to 75.9% (at 744 h) according to the chemical composition of the LDH precursors, and the use or not of CMC. It is a good point for versatile production, since other LDH compositions or biopolymers in the form of powder or plasticizers may be tested, depending on the expected release properties.

## 4. Conclusions

In this study, using the mechanochemical reaction between calcined LDH and K_2_HPO_4_ to prepare SRF, promising results were obtained with respect to the slow release behavior of P and K. The studies scrutinized the effect of the temperature of LDH calcination, showing that a thermal treatment at 200 °C is suitable to produce materials for the grinding process which display satisfactory release results. Higher calcination temperatures promote phosphate precipitation for longer release times (168 h), making compounds unsuitable as SRF. The release behavior for both systems studied is strongly dependent on the LDH chemical composition and on the temperature of the medium. Interestingly, in the presence of LDH precursors, even if struvite-K crystallizes after milling, and at short release time, it subsequently dissolves and it is probable that this formed structure is strongly related to the slow-release character. Furthermore, the addition of carboxymethylcellulose allows tuning of the release properties enhancing the release percentage of phosphate. This study involving LDH matrices allows the development of the SRF production process. The nature of the starting synthetic LDH precursors applied in mechanochemical assays enables a distinct study compared to systems involving natural precursors, such as clay minerals and LDH, ion exchange studies. The efficiency of these LDH based SRF on plant growth are under investigation to confirm their relevance in real-world conditions.

## Figures and Tables

**Table 1:** Description of the three methods used to incorporate carboxymethylcellulose (CMC) into the systems’ layered double hydroxides (LDH) matrices calcined at 200 °C.

System	Method	Time of Milling (h)	LDH/K_2_HPO_4_/CMC Weight Ratio
MgAl/K_2_HPO_4_/CMC	Method 1	3	1.00:1.65:0.53
	Method 2	3	1.00:1.65:0.53
	Method 3	9	1.00:1.65:0.53
MgFe/K_2_HPO_4_/CMC	Method 1	3	1.00:0.82:0.40
	Method 2	3	1.00:0.82:0.40
	Method 3	9	1.00:0.82:0.40

**Table 2:** Kinetic data for MgAl_200_/K_2_HPO_4_ material.

Nutrient	Temperature (°C)	Kinetic Parameters			
K		Pseudo-second order			
		*k_II_* (min^−1^)	*q_e_* (mg g^−1^)	*R* ^2^	Exp. *q_e_* (mg g^−1^)
	10	0.070	1.32	0.997	1.31
	25	0.134	1.46	0.999	1.44
	50	0.519	1.43	0.999	1.44
P	10	0.085	0.722	0.995	0.717
	25	0.342	0.462	0.999	0.453
	50	0.481	0.738	0.999	0.733

Note: *q_e_* = desorbed ion at equilibrium time, *k_II_* and *k_d_* = kinetic constant.

**Table 3:** Kinetic data for MgFe_200_/K_2_HPO_4_ material Table 3.

Nutrient	Temperature (°C)	Kinetic Parameters						
K		Pseudo-second order						
		*k_II_* (min^−1^)	*q_e_* (mg g^−1^)			*R* ^2^	Exp. *q_e_* (mg g^−1^)	
	10	0.31	0.447			0.998	0.443	
	25	1.06	0.594			0.999	0.592	
	50	0.631	0.542			0.999	0.541	
P		Intraparticular diffusion						
		*K_d_* (mg g^−1^ min^−0.5^)		Intercept				*R* ^2^
	10	0.001		0.002				0.993
	25	0.003		0.001				0.995
		Pseudo-second order						
		*k_II_* (min^−1^)	*q_e_* (mg g^−1^)		*R* ^2^		Exp. *q_e_* (mg g^−1^)	
	50	1.79	0.0826		0.997		0.0807	

Note: *q_e_* = desorbed ion at equilibrium time, *k_II_* and *k_d_* = kinetic constant.

**Table 4:** Percentage release of the elements for CMC/MgAl_200_/K_2_HPO_4_ and CMC/MgFe_200_/K_2_HPO_4_ composites.

Release time	CMC/ MgAl_200_/K_2_HPO_4_			CMC/MgFe_200_/K_2_HPO_4_	
		P (%)	K (%)	P (%)	K (%)
1 h	Method 1	6.6	16.3	2.4	12.9
	Method 2	8.0	23.0	6.2	25.4
	Method 3	11.1	21.7	5.9	17.1
168 h	Method 1	73.3	100.0	33.4	82.7
	Method 2	62.3	92.6	28.2	80.6
	Method 3	69.5	92.4	32.0	76.4

## Supplementary Materials

The following are available online at http://www.mdpi.com/2079-4991/9/2/183/s1, Figure S1: FTIR data of LDH precursors and calcined LDH; Figure S2: FTIR data of milled samples; Figure S3: PXRD of milled samples obtained with CMC; Figure S4: ^13^C NMR of milled samples obtained with CMC; Figure S5: ^27^Al and ^31^P NMR of CMC/MgAl_200_/K_2_HPO_4_; Figure S6: SEM images of milled samples obtained with CMC; Table S1: XRD data for struvite-K; Table S2: ^13^C NMR milled samples obtained with CMC.
