# Supplementary material for: Potential Sustainable Slow-Release Fertilizers Obtained by Mechanochemical Activation of MgAl and MgFe Layered Double Hydroxides and K2HPO4
Source: Nanomaterials (Basel). 2019 Feb 1;9(2):183. doi: 10.3390/nano9020183 (PMC6410234; doi:10.3390/nano9020183)
Supplement: Supplementary file 1 [file nanomaterials-09-00183-s001.pdf]

Supplementary materials

Potential sustainable slow-release fertilizers obtained by mechanochemical activation of MgAl and MgFe layered double hydroxides and K_2HPO_4.

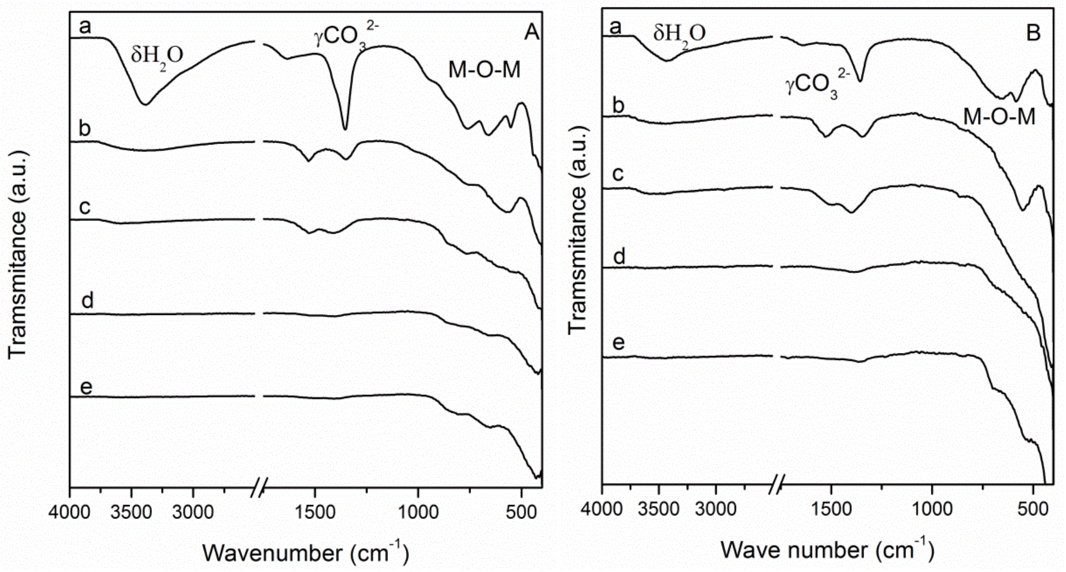

Figure S1. FTIR data of A) $Mg_2Al(OH)_6(CO_3).3H_2O$ (a), MgAl calcined at: 200 °C (b); at 300 °C (c); at 400 °C (d) and at 500 °C (e) and B) $(Mg_2Fe(OH)_6(CO_3).2.5H_2O$ (**a**), MgFe calcined at: 200 °C (b); at 300 °C (c); at 400 °C (d); and at 500 °C (e).

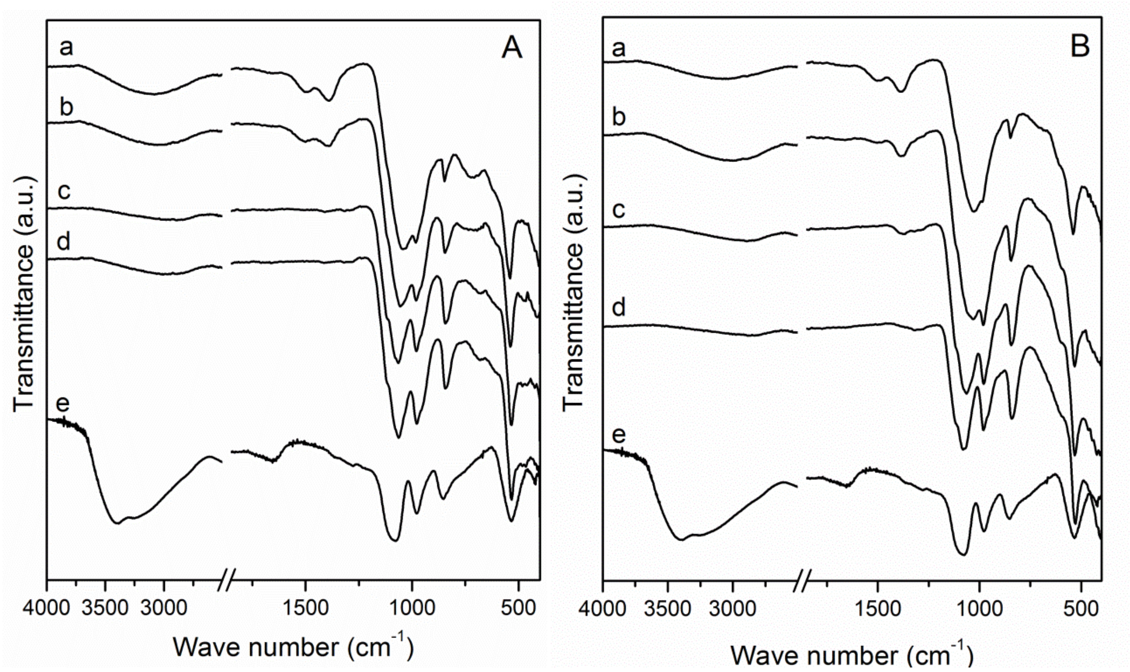

Figure S2: FTIR data of milled samples (9 h, 450 rpm) A) $MgAl_{200}/K_2HPO_4$ (a); $MgAl_{300}/K_2HPO_4$ (b); $MgAl_{400}/K_2HPO_4$ (c); $MgAl_{500}/K_2HPO_4$ (d), K_2HPO_4 (e), and B) $MgFe_{200}/K_2HPO_4$ (a); $MgFe_{300}/K_2HPO_4$ (b); $MgFe_{400}/K_2HPO_4$ (c); $MgFe_{500}/K_2HPO_4$ (d), K_2HPO_4 (e) with LDH/K_2HPO_4 MR 1:2.

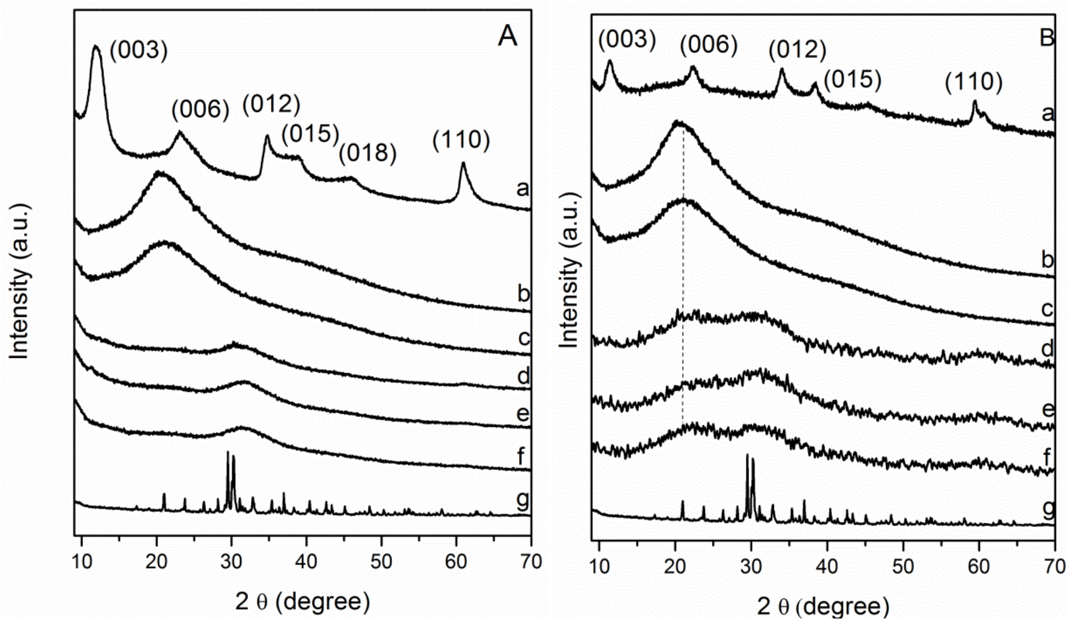

Figure 3. PXRD for A) MgAl (a), CMC (b), CMC milled for 3 h (c) and CMC/$MgAl_{200}/K_2HPO_4$ (Method 1) (d), CMC/$MgAl_{200}/K_2HPO_4$ (Method 2) (e), CMC/$MgAl_{200}/K_2HPO_4$ (Method 3) (f), K_2HPO_4 (g) and B) MgFe (a), CMC (b), CMC milled for 3 h (c), CMC/$MgFe_{200}/K_2HPO_4$ (Method 1) (d), CMC/$MgFe_{200}/K_2HPO_4$ (Method 2) (e) and CMC/$MgFe_{200}/K_2HPO_4$ (Method 3) (f) and K_2HPO_4 (g).

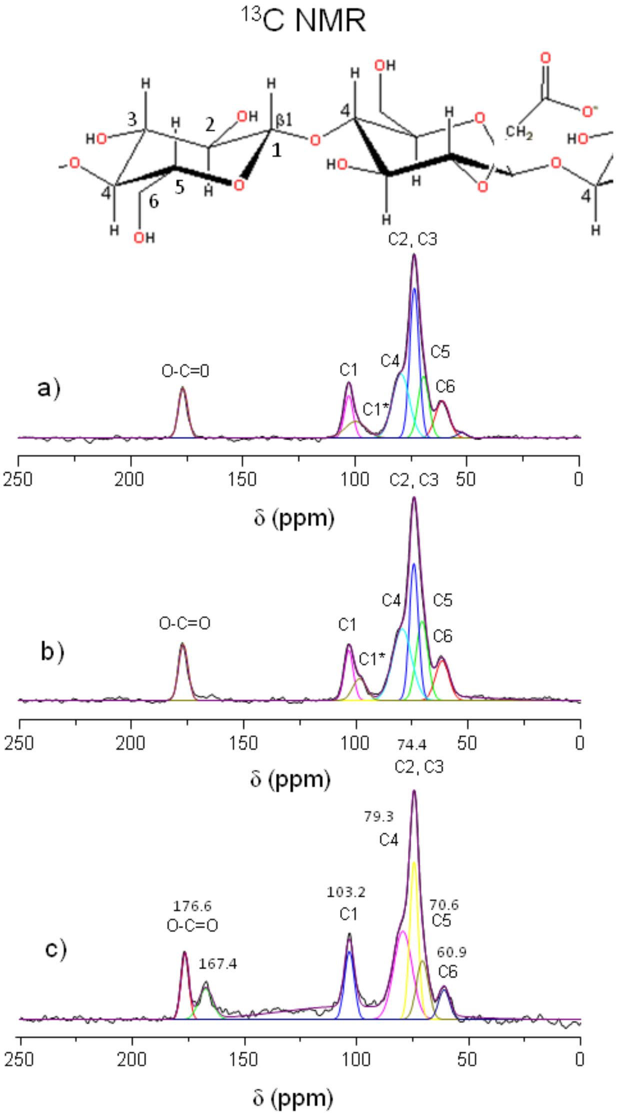

Figure. S4: ^{13}C NMR of CMC (**a**); CMC milled (**b**); and CMC/$MgAl_{200}$/K_2HPO_4 (Method 2) (**c**).

.

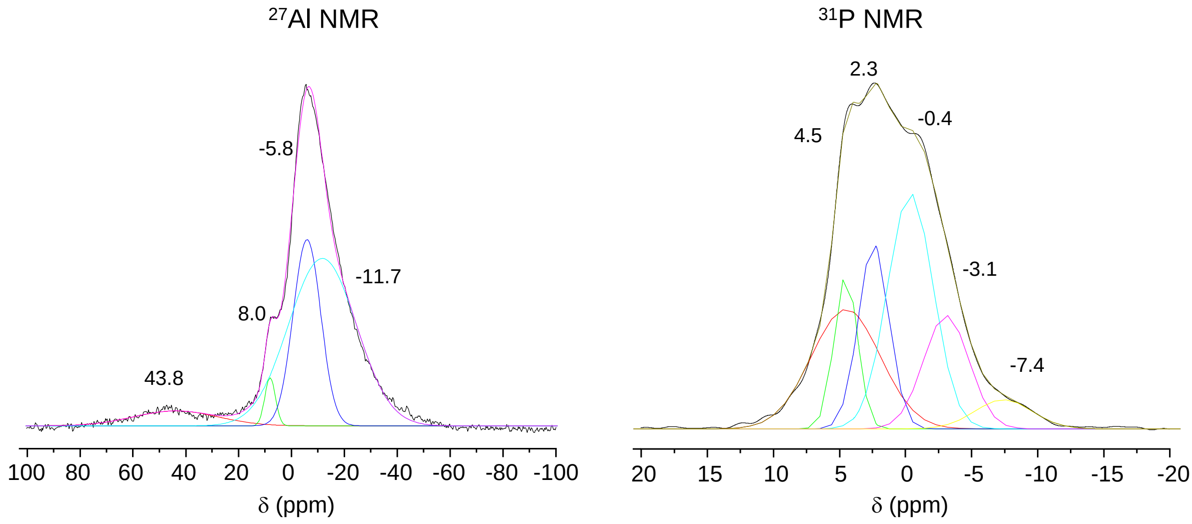

Figure. S5: ^{27}Al and ^{31}P NMR of CMC/$MgAl_{200}$/K_2HPO_4 (Method 2).

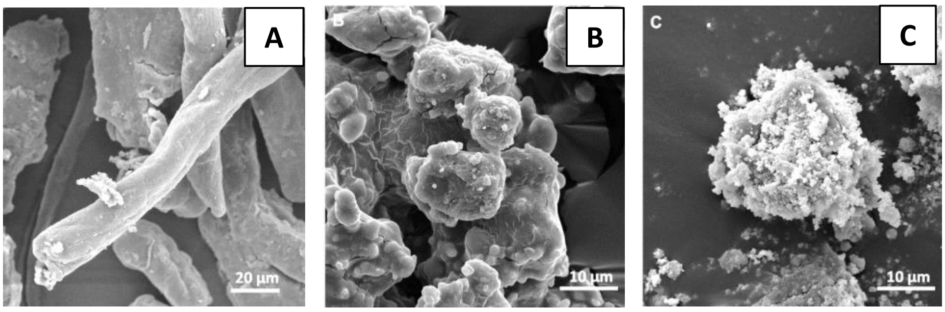

Figure. S6: SEM images of CMC (**A**), CMC milled with: $MgAl_{200}$/K_2HPO_4 (**B**), and $MgFe_{200}$/K_2HPO_4 (**C**).

Table S1: XRD data for struvite-K identified in $MgAl_{200}$/K_2HPO_4.

2θ (°)	d_{hkl} (Å)	hkl
21,06	4,218	111
21,70	4,095	012
26,02	3,424	112
27,50	3,243	103
31,02	2,882	211
32,40	2,763	004
33,38	2,684	022
34,12	2,627	212

Table S2. ^{13}C NMR of CMC, CMC milled, $CMC/MgAl_{200}/K_2HPO_4$ (Method 2).

Site	sample	δ (ppm)	I(%)
C=0	CMC	177.0	10.0
	CMC_{milled}	176.9	10.9
	$CMC/MgAl_{200}/K_2HPO_4$	176.6	9.7
C1	CMC	103.2	9.9
	CMC_{milled}	103.2	10.8
	$CMC/MgAl_{200}/K_2HPO_4$	103.2	11.8
C1*	CMC	100.1	23.5
	CMC_{milled}	98.4	15.8
	$CMC/MgAl_{200}/K_2HPO_4$	-	-
C2-C3	CMC	74.0	10.2
	CMC_{milled}	72.2	10.4
	$CMC/MgAl_{200}/K_2HPO_4$	74.4	10.4
C4	CMC	80.0	18.9
	CMC_{milled}	79.4	22.2
	$CMC/MgAl_{200}/K_2HPO_4$	79.3	22.5
C5	CMC	69.9	12.0
	CMC_{milled}	70.6	13.7
	$CMC/MgAl_{200}/K_2HPO_4$	70.6	14.8
C6	CMC	61.5	15.5
	CMC_{milled}	61.6	16.0
	$CMC/MgAl_{200}/K_2HPO_4$	60.9	13.2
C=O*	$CMC/MgAl_{200}/K_2HPO_4$	167.4	17.5
